# Multi-Omics on Traditional Medicinal Plant of the Genus *Aconitum*: Current Progress and Prospect

**DOI:** 10.3390/molecules30010118

**Published:** 2024-12-31

**Authors:** Ting Wang, Cai Rangji, Wenbin Liu, Jing Ma, Ruichen Zhou, Liang Leng, Yi Zhang

**Affiliations:** 1 State Key Laboratory of Southwestern Chinese Medicine Resources, Chengdu University of Traditional Chinese Medicine, Chengdu 611137, China; wangting19981213@163.com (T.W.); cairangji@stu.cdutcm.edu.cn (C.R.); liuwenbin@stu.cdutcm.edu.cn (W.L.); ponymajing@stu.cdutcm.edu.cn (J.M.); zrc1104@163.com (R.Z.); 2 Ethnic Medicine Academic Heritage Innovation Research Center, School of Ethnic Medicine, Chengdu University of Traditional Chinese Medicine, Chengdu 611137, China; 3 Institute of Herbgenomics, Chengdu University of Traditional Chinese Medicine, Chengdu 611137, China; 4 School of Pharmacy, Chengdu University of Traditional Chinese Medicine, Chengdu 611137, China

**Keywords:** *Aconitum*, genomics, transcriptomics, metabolomics, proteomics, microbiomics, multi-omics

## Abstract

*Aconitum* stands out among the Ranunculaceae family for its notable use as an ornamental and medicinal plant. Diterpenoid alkaloids (DAs), the characteristic compounds of *Aconitum*, have been found to have effective analgesic and anti-inflammatory effects. Despite their medicinal potential, the toxicity of most DAs restricts the direct use of *Aconitum* in traditional medicine, necessitating complex processing before use. The use of high-throughput omics allows for the investigation of *Aconitum* plant genetics, gene regulation, metabolic pathways, and growth and development. We have collected comprehensive information on the omics studies of *Aconitum* medicinal plants, encompassing genomics, transcriptomics, metabolomics, proteomics, and microbiomics, from internationally recognized electronic scientific databases such as Web of Science, PubMed, and CNKI. In light of this, we identified research gaps and proposed potential areas and key objectives for *Aconitum* omics research, aiming to establish a framework for quality improvement, molecular breeding, and a deeper understanding of specialized metabolite production in *Aconitum* plants.

## 1. Introduction

The genus *Aconitum* belongs to the family Ranunculaceae of the Angiospermae and includes annual or perennial herbs [1]. It consists of more than 350 species worldwide, primarily located in temperate regions of the Northern Hemisphere [2]. Asia and Europe are the regions with the highest concentration of *Aconitum* species, with over 200 different species being present in China. *Aconitum* plants thrive in the Hengduan Mountains, linking Sichuan, Yunnan, and Tibet, suggesting that this region is a hotspot for *Aconitum* evolution [3]. Additionally, the Himalayas are a key contributor to the abundance of species in multiple Asian countries, including India, Bhutan, Nepal, and others, where *Aconitum* plants can be observed [4]. A total of 94 species of *Aconitum* plants have been recorded in Europe, including varieties and naturally hybridized plants [5]. The *Aconitum* genus is known for being ornamental and possesses distinct biological traits, including vibrant hues, unusual flower shapes, and the ability to withstand cold temperatures, making them a valuable source of blue flower genetics [6].

Various traditional applications and therapeutic effects of *Aconitum* plants have been recorded in some Asian countries through prolonged contact and usage [7]. This illustrates the significance of *Aconitum* plants in Asian herbal medicine. The genus *Aconitum* is divided into three subgenera: subgen. *Lycoctonum*, subgen. *Aconitum*, and subgen. *Gymnaconitum* [8]. Among them, *Aconitum* medicinal plants are mainly derived from the subgenus *S. Aconitum*. There are approximately 70 species of *Aconitum* plants used medicinally, including well-known Chinese healing herbs such as Chuanwu (*Aconitum carmichaelii*), Fuzi (*Aconitum carmichaelii*), and Caowu (*Aconitum kusnezoffii*) [9]. *Aconitum* plants contain alkaloids, flavonoids, steroids, glycosides, and other chemical components [10], which play important roles in anti-inflammatory, anti-tumor, analgesic, and insecticidal activities [11]. A wide variety of conditions can be treated with them, including rheumatoid arthritis, inflammatory diseases, pain, trauma and fractures, poisoning, colds, and immunosuppressive diseases [12]. Notwithstanding their long history as medicinal plants, some species of *Aconitum* are also well known as toxic plants. Poisons derived from them have been used as powerful poisons since ancient times, such as arrow poisons and fish bait poisons [13]. Improper processing or ingestion of *Aconitum* drugs may result in poisoning cases manifested as damage to the heart and nervous system, seriously threatening physiological health and life safety [14]. Owing to the highly toxic nature of *Aconitum* medicinal materials, various processing methods and techniques, such as boiling and steaming, have been historically developed to achieve the aim of “reducing toxicity while maintaining efficacy” [15,16]. Studies have found that the therapeutic value of *Aconitum* medicinal plants is due to their high content of C_19_-type diterpenoid alkaloid compounds [17]. However, their toxicity has limited the widespread use of DAs of the C_18_ and C_19_ types [18]. According to quantitative structure–activity relationships and toxicological studies, the toxicity of DAs is closely related to the acetyl group in the C-8 position and the benzoyl group in the C-14 position in their skeletons [19]. Research on the relationship between the significant pharmacological effects and high toxicity of *Aconitum* alkaloids and their structures is an important topic for scholars from both China and other countries [20,21,22]. Among the 46 known DA skeletons, 40 are present in the Ranunculaceae family, and approximately half of these DAs are derived from *Aconitum* plants. *Aconitum* plants offer a promising model for the study of DA biosynthesis due to the diversity of DA species and their diverse biological activities [23].

The development of molecular sequencing technologies has substantially improved our knowledge of the enzymes and metabolic pathways that lead to the evolution of particular secondary metabolites [24]. Agricultural, environmental, and pharmaceutical scientists are interested in *Aconitum* plants for their resource value, ornamental value, and medicinal value. Global trade of different *Aconitum* species is increasing due to a large market in Asia and Europe, which leads to the indiscriminate harvesting of many *Aconitum* species in the wild [25]. The International Union for Conservation of Nature (IUCN) has listed some *Aconitum* species, such as *A. heterophyllum* and *A. corsicum*, as threatened due to the rapid decline of wild populations [26].

**Figure 1:**
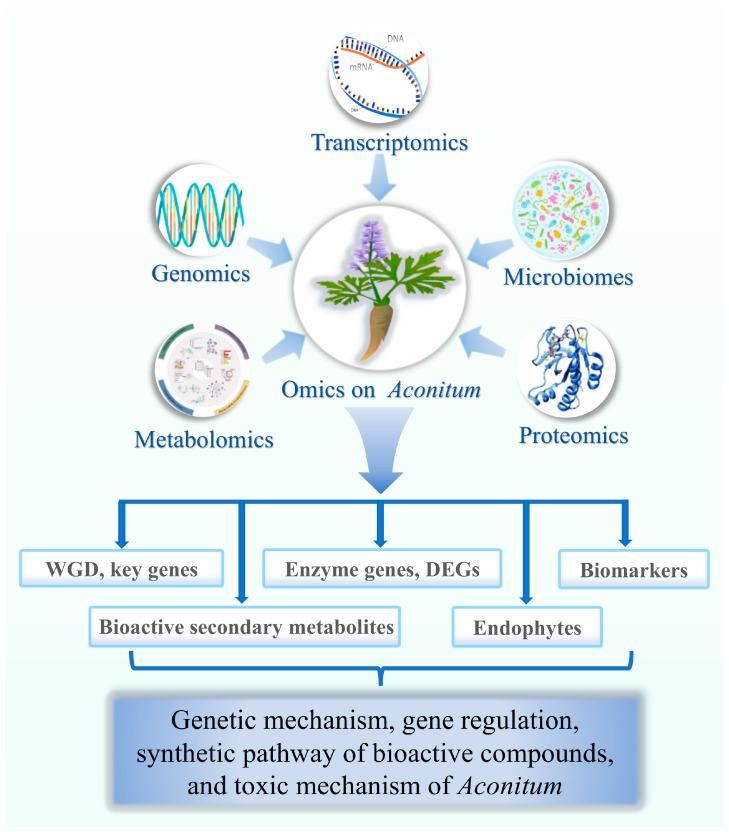
Application of multi-omics in *Aconitum*. Integrating data from different levels (genes, metabolites, proteins, and endophytes) can make it possible to make feasible and reliable discoveries of potentially key factors responsible for bioactive compounds, as well as physiological processes and characteristics. WGD (genome-wide duplication event); DEGs (differentially expressed genes).

Therefore, in order to genetically intervene to increase the expression of targeted metabolites and obtain DA components, elucidating the biosynthesis pathway of *Aconitum* DAs is necessary. This is vital for sustainable *Aconitum* resource utilization. The use of genomics, transcriptomics, proteomics, metabolomics, and other omics has become prevalent in medicinal plant research thanks to advancements in detection techniques and analytical tools [27,28]. The application of omics technologies can be helpful in analyzing the chemical structure, metabolic pathway, and gene regulation pattern of active secondary metabolites, which provides researchers with the opportunity to better understand plant natural product biosynthesis, analyze the genes and pathways involved in plant primary metabolite biosynthesis, and provide theoretical and technical support for developing and utilizing plant resources [29]. Therefore, the omics method has great potential in *Aconitum*. Previous reviews on *Aconitum* species have primarily focused on their genetic diversity and biosynthetic pathways of active components [13,26]. This review focuses on the latest research progress in *Aconitum* based on various omics (Figure 1), including previously unreviewed research areas such as genomics, proteomics, and microbiome studies. Additionally, we discuss the limitations of the current study. Finally, a prospective topic for the further study of *Aconitum* medicinal plants has been put forward.

## 2. Genomic Research in *Aconitum*

*Aconitum* species have adapted to changing environments and other factors through karyotype evolution, such as polyploidization [30,31]. Most of these plants have eight chromosome pairs. *A. fletcheranum* is a unique *Aconitum* species with six chromosome pairs (2n = 12) [32], while others belong to diploid (2n = 16), tetraploid (2n = 32) [33], and hexaploid (2n = 48) categories; *A. apetalum* is the hexaploid plant with the highest ploidy level within the genus [5] (Table 1). Due to the polyploidization of *Aconitum* plants with highly heterozygous genomes, it has been challenging to assemble and sequence the genomes. Consequently, there has been a lack of genomic information for *Aconitum* plants. Recently, the genome of the first medicinal plant of the *Aconitum* genus, *A. vilmorinianum* Kom. (2n = 16), was reported [34]. This assembly has a size of 5.76 Gb, a contig N50 value of 311.29 kb, a heterozygosity of 0.31%, and a repeat content of 80.52%. Notably, through comparative genomic analysis, a WGD specific to *A. vilmorinianum* was discovered. The findings may provide a deeper understanding of DA biosynthesis pathways and the evolutionary mechanisms involved. In summary, parsing the genomic information of important medicinal plants through genomics sequencing, such as desirable traits and biosynthetic pathways of effective compounds, will become a future trend in improving or cultivating medicinal plant varieties.

Due to their various species and natural environmental changes, *Aconitum* species are difficult to reliably identify based on just their physical traits [1]. However, recent advances in chloroplast genome sequencing technology have brought breakthroughs to the identification and evolutionary studies of *Aconitum* [35]. In plants, chloroplasts are unique organelles that possess a complete genome structure, conserved sequences, and abundant mutation sites, which make them essential for the identification of species and the study of evolution [36]. The chloroplast genomes of *Aconitum* plants exhibit a typical circular quadripartite structure, ranging in size from 155,000 to 157,000 base pairs, with similar gene content and GC content. The large single-copy (LSC) region and the small single-copy (SSC) region are separated by two inverted repeat (IR) regions, and the IR region is more conserved than the LSC and SSC; its high conservation helps to maintain the basic function and structure of chloroplasts. *Aconitum* chloroplast genome analysis provides a powerful tool for identifying species and studying evolutionary relationships by revealing the complex phylogenetic relationships among plants [37]. A comparative overview of chloroplast genome sequencing in the past ten years is summarized in Table 2.

## 3. Transcriptomic Research in *Aconitum*

**Figure 2:**
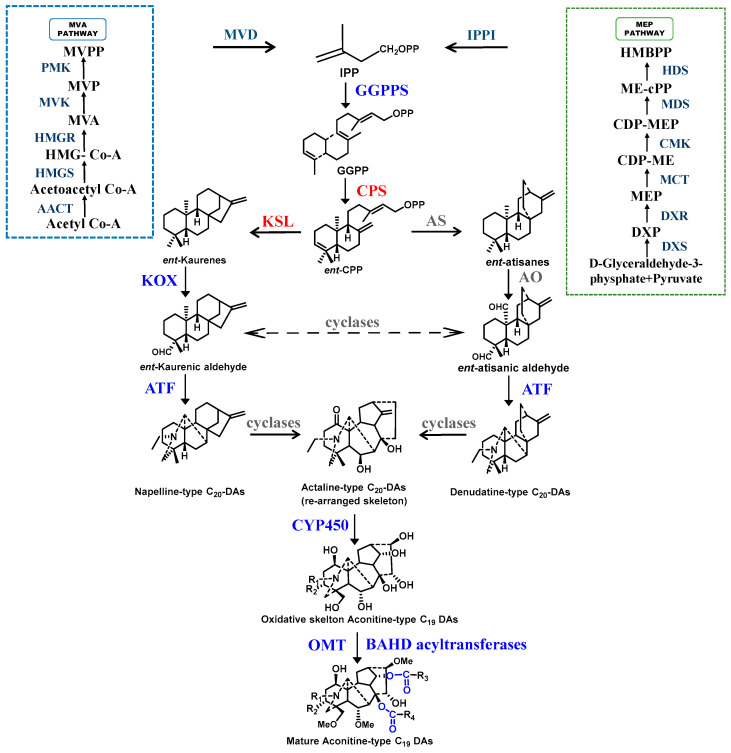
Schematic of the putative biosynthetic pathway of DAs in *Aconitum* plants. The precursors for DA biosynthesis are generated from the Methylerythritol (MEP) and Mevalonate (MVA) pathways. The specific genes within the red-marked enzyme families have been identified, while those marked in gray remain undiscovered, and the enzyme genes marked in blue are predicted from transcriptome sequencing but have not been experimentally validated. DA skeleton rearrangement: cyclases and CYP450; skeleton decoration: CYP450, OMT, and BAHD acyltransferases. Abbreviations: AACT (acetoacetyl-CoA thiolase), HMGR (3-hydroxy-3-methylglutaryl-CoA reductase), HMGS (3-hydroxy-3-methylglutaryl-CoA synthase), MVK (mevalonate kinase), PMK (phosphomevalonate kinase), MVD (mevalonate diphosphate decarboxylase), DXS (1-deoxy-D-xylulose 5-phosphate synthase), DXR (1-deoxy-D-xylulose 5-phosphate reductoisomerase), MCT (2-C-methyl-D-erythritol-4-phosphate cytidylyltransferase), CMK (4-diphosphocytidyl-2-C-methyl-D-erythritol kinase), MDS (2-C-methyl-D-erythritol 2,4-cyclodiphosphate synthase), HDS ((E) -4-hydroxy-3-methylbut-2-enyl-diphosphate synthase )IPPI (isopentenyl diphosphate isomerase), GGPPS (geranylgeranyl pyrophosphate synthase), CPS (*ent*-copalyl diphosphate synthase), KSL (*ent*-kaurene synthase-like), KOX (*ent*-Kaurene oxidase), AS (*ent*-atisane synthase), AO (*ent*-atisane oxidases), ATF (L-serine aminotransferases), CYP450 (Cytochrome P450 monooxygenase), and OMT (O-methyltransferase).

Transcriptomics refers to the study of all RNA transcribed by cells at a specific time and in a specific space, presenting the transcription status of the genes contained within the cells and thereby uncovering the regulatory patterns of their transcription [13]. Transcriptome sequencing has developed rapidly, making high-throughput sequencing one of the most widely used tools for studying biosynthetic genes of secondary metabolites [52]. The transcriptional characteristics of *Aconitum* species, including *A. carmichaelii* [53], *A. heterophyllum* [54], *A. gymnandrum* [55], and *A. vilmorinianum* [56], have been reported, and numerous candidate genes involved in DA biosynthesis have been predicted [57]. DA biosynthesis mainly involves three processes: the production of diterpenoid precursors, the formation of the DA skeleton by inserting N atoms into the diterpenoid precursors, and the modification of the DA skeleton [23]. Isoprenoid (IPP) is the end product of both the Mevalonate (MVA) and the Methylerythritol (MEP) pathways and serves as the precursor for DAs [58,59]. By means of transcriptome analysis, a large number of enzyme genes involved in the synthesis of diterpene precursors, including those related to the MVA and MEP pathways, were identified. There are also a few unigenes regulating DA biosynthesis that were identified, including geranylgeranyl pyrophosphate synthase (GGPPS), which condenses IPP molecules to form geranylgeranyl pyrophosphate (GGPP); *ent*-copalyl diphosphate synthase (CPS), responsible for converting GGPP into *ent*-copalyl diphosphate (*ent*-CPP); and kaurene synthase (KS), which synthesizes *ent*-kaurene [53]. Subsequently, enzymes encoding the formation and modification of the DA skeleton were identified through next-generation sequencing of rootstocks and leaf tissues of *A. carmichaelii* [60]. These enzymes include kaurene oxidases (KOX) that catalyze the formation of aldehyde groups, cyclases that interconvert the *ent*-kaurene or *ent*-atiserene skeletons, and key aminotransferases that act as the nitrogen source of DA to form the C_20_ diterpene alkaloid skeleton [61], as well as monooxygenases, O-methyltransferase (OMT), and BAHD acyltransferases (named after the initial letters of the four enzymes whose biochemical functions were first identified in this family, namely benzylalcohol O-acetyl transferase (BEAT), anthocyanin O-hydroxycinnamoyl transferases (AHCTs), anthranilate N-hydroxycinnamoyl/benzoyltransferase (HCBT), and deacetylvindoline 4-O-acetyltransferase (DAT)), which catalyze the modification of the DA skeleton to form structurally diverse DAs (Figure 2). Among them, it was found in *A. vilmorinianum* that hydroxymethylglutaryl-CoA reductase (HMGR) is a critical regulatory enzyme in MVA metabolism and is regulated by a microRNA mir6300 [56]. 1-Deoxy-D-xylulose-5-phosphate synthase (DXS) functions as a blocking enzyme in the MEP pathway [54]. KS and CPS play key roles in DA biosynthesis, and CPS accumulates DAs specific to different tissues [53]. In addition, not only enzymes encoding diterpene biosyntheses such as KOX and kaurenoic acid hydroxylase (KH) but also other potential genes related to the process of biosynthesis of atisine were discovered in *A. heterophyllum*, including those related to glycolysis and serine biosynthesis [62].

By looking at the transcriptome of various tissue types, it has been found that certain tissues have a specific buildup of bioactive compounds, with the enzymes and transcription factors responsible for this also displaying tissue-specific expression patterns [63,64,65,66]. As reported for the tuberous roots of *Aconitum* that store secondary metabolites, an analysis of the transcriptomic data of *A. heterophyllum* has identified that eight genes encoding GDP-mannose pyrophosphory-lase (GMPase), SRF receptor kinase (SRF), RING-box protein 1 (RBX1), ADP glucose pyrophosphorylase (AGPase) and auxin-responsive factor 2 (ARF2) are highly expressed in roots, indicating that they may influence root growth and have the capability to boost supplies of bioactive compounds [67]. By analyzing the transcriptomic data of *A. kusnezoffii*, the mechanisms for distributing resources among vegetative propagation, sexual reproduction, and defense throughout growth were investigated. It was found that the main root serves as a storage and resource allocation center for photosynthetic products, and the buildup of chemicals and poisons in asexual propagules was assured by the consumption and movement of resources from the main root. After flower formation, plants start to amass protective molecules, which may be utilized again for additional processes [68]. In *A. kusnezoffii*, the MVA pathway is more dominant in flowers than the MEP pathway, whereas it is the opposite in leaves and stems. GGPPS is relatively highly expressed in flower and leaf tissues. CPS and KS are relatively more active in stems, while most genes are expressed relatively lowly in root tissues. It is speculated that flowers may be the main biosynthetic source of aconitine alkaloids, while lateral roots serve as storage sites, indicating there exists a source–sink relation [69]. Most of the flowers in the genus *Aconitum* are blue. A study found that during the development of the flowers, the expression level of the flavonoid 3,5′-hydroxylase gene named Av-F3′5′H increased gradually, reaching its peak when the flowers turned completely blue. It is speculated that this gene may be involved in the formation of the blue flowers of *Aconitum* and plays an important regulatory role [70]. Transcriptomic data can be leveraged to improve the genetics of *Aconitum* species, thereby enhancing their biomass or secondary metabolites.

Through their varied and spatial actions on target genes, transcription factors (TFs) have a significant impact on the growth and development of plants, as well as their stress response [71,72]. Currently, many TFs regulating the synthesis and accumulation of bioactive compounds have been discovered in various *Aconitum* medicinal plants through transcriptomic data, mainly including MYB, WRKY, C3H, ERF, bHLH, FAR1, bZIP transcription factor families, and ABC transporters. Among them, the MYB family has the largest number of genes [53,54,56,69]. On the other hand, terpene synthesis consists of two principal enzyme families, including terpene synthases and cytochrome P450s. In *A. carmichaelii*, DA is produced solely by the combination of TPS-c and TPSe/f subfamily genes [73]. The cytochrome P450 family, an enzyme family that promotes the diversity of DA molecules, has been identified in several transcriptome investigations as a potential candidate gene implicated in secondary metabolite biosynthesis. The main CYP450 genes identified belong to the CYP71 family and the CYP88A1 family [53,56]. BAHD acyltransferases act as terminal enzymes catalyzing the synthesis of DAs, modifying secondary metabolites by acylation to form esters [74]. These enzymes may be involved in the biosynthesis of highly toxic DAs. Members of clade III in the BAHD phylogenetic tree utilize acetyl-CoA as the main acyl donor and participate in the acylation modification of alkaloids, indicating that this clade should be responsible for the formation of acetyl esters at the C-8 position [75]. Members of the V_a_ subfamily utilize hydroxycinnamoyl/formyl CoA as acyl donors, and it is speculated that members of Va are responsible for decorating the C-14 position of DA to form benzoyl esters [76]. BAHD acyltransferases from both branches may be involved in the biosynthesis of C_19_-DA from *A. carmichaelii*, indicating a close association with *Aconitum* toxicity [60].

Microsatellites (SSRs) are DNA regions that are widely distributed throughout the genome of plants and are prone to mutation. There is wide use of SSRs as molecular markers in selective breeding, genetic diversity assessment, and the study of genetic variation in populations [77]. Based on 56,692 root transcripts and 32,719 stem transcripts from *A. heterophyllum*, 177,438 and 118,814 potential SSRs were identified [54]. Among the SSRs identified in the *A. carmichaelii* transcriptome, there were 63.68 percent mononucleotide repeats among all SSRs. Similarly, *A. heterophyllum* also had the highest proportion of mononucleotide repeats (61%), followed by trinucleotide (25.37%) and dinucleotide (12.42%) repeats [56]. The main type of SSR in the root transcriptome of *A. vilmorinianum* was trinucleotide, with AAG/CTT being the most common [56]. These potential SSRs will provide rich resources for the development of SSRs in this important medicinal plant species, and more *Aconitum* species are needed to participate in the construction of genetic information for *Aconitum* plants.

Until now, only partial CPS and kaurene synthase-like (KSL) genes of terpene synthase (diTPS) from *A. carmichaelii* and *A. gymnandrum* have been functionally identified [55,73]; AcCPS1, AcCPS2s, AcKSL1, AcKSL2s, and AcKSL3-1 appear to be responsible for all the known C_20_-DAs biosynthesis in *A. carmichaelii*, and C_18_ and C_19_ DAs may be derived from the C_20_-type kaurane and atisane, as suggested by the transcriptome data [73]. Functional identification revealed that AgCPS1, AgCPS2, and AgCPS4 can catalyze GGPP to produce *ent*-CPP; in addition to *ent*-CPP, researchers discovered *ent*-8,13-CPP as a potential precursor to DA synthesis. AgCPS5 can catalyze GGPP to produce *ent*-8,13-CPP, which, when coupled with AgKSL1, generates *ent*-atiserene in *A. gymnandrum* [55]. Even though many transcriptome studies and genes are associated with the biosynthesis of active components in *Aconitum* plants, the number of genes identified is far from sufficient. More transcriptome studies and functional gene identification in different *Aconitum* plants are needed to provide a comprehensive gene library and theoretical basis for the biosynthetic pathway of DAs. Finally, further exploration is required to understand the involvement of different CYP450s, methyltransferases, BAHD acyltransferases, and additional enzymes in the structural alteration of *Aconitum* DAs for their variety.

## 4. Metabolomics Research in *Aconitum*

**Figure 3:**
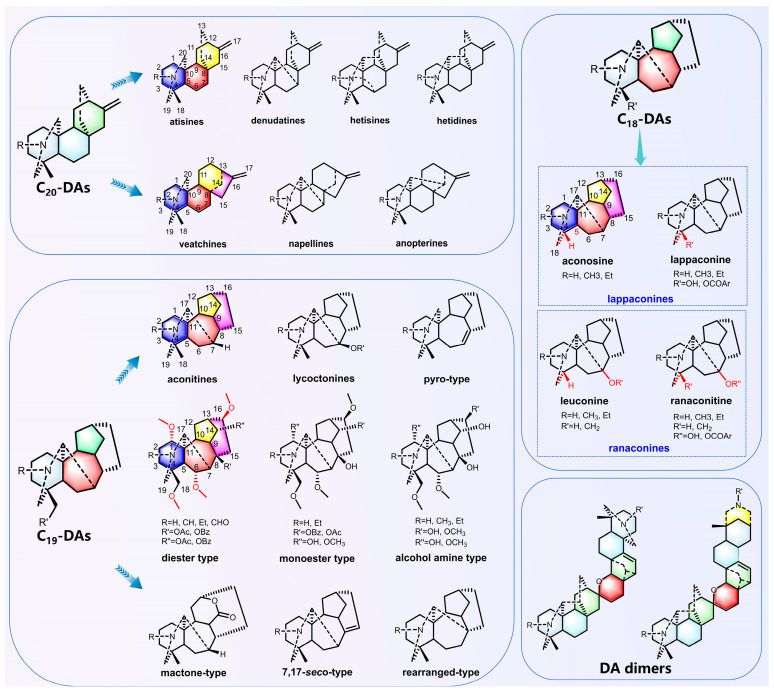
Classification of DA structures in the genus *Aconitum*. DAs are classified into C_20_-DAs, C_19_-DAs, C_18_-DAs, and DA dimers based on the number of carbon atoms and chemical structure types. The carbon numbering for the typical chemical structure is labeled in the figure.

**Figure 4:**
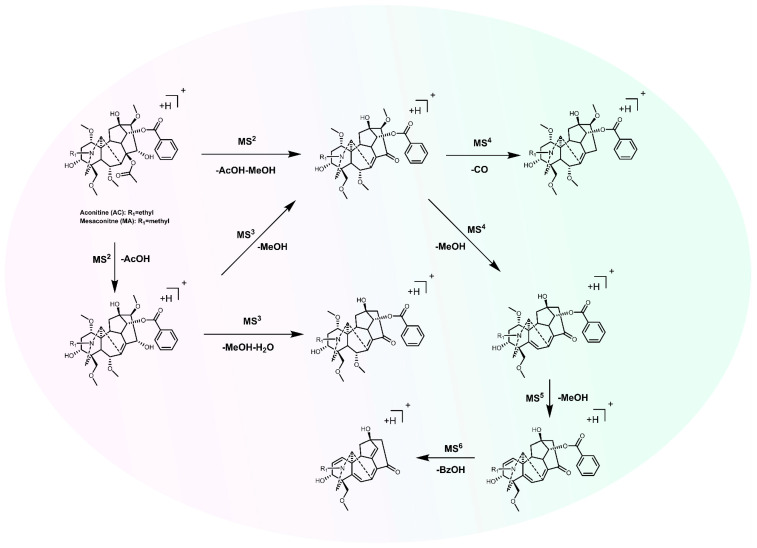
The mass spectrum fragmentation pattern of aconitine.

Metabolites, especially secondary metabolites in plants, reflect the plants’ responses to environmental changes [78]. Although the metabolites in medicinal plants are influenced by various factors, such as genetics, environment, growth, and development, resulting in a complex array of metabolites, the high-throughput methods of metabolomics enable the rapid identification and analysis of a large number and variety of metabolites, thereby elucidating the regulatory network of effective substance synthesis in medicinal plants. In the genus *Aconitum*, the most typical compounds are DAs, which are generally classified into C_20_-DAs, C_19_-DAs, C_18_-DAs, and DA dimers based on the number of carbon atoms and chemical structural types (Figure 3). C_20_-DAs possess complex structures, with most of them containing exocyclic double bonds. Their structures can be categorized into types such as atisines, denudatines, hetisines, hetidines, veatchines, napellines, and anopterines. Most C_20_-DAs do not contain methoxy groups in their structures, whereas almost all C_18_-DAs and C_19_-DAs possess methoxy groups. C_19_-DAs, which have evolved from C_20_-DAs, are among the most abundant and highly toxic plant components discovered to date. Identified in *Aconitum*, they predominantly feature the aconitine skeleton and are consequently frequently designated as aconitine-type alkaloids. The mass spectrum fragmentation pattern of aconitine is illustrated in Figure 4. According to the carbon skeleton and specific substituents at certain positions of the C_19_-DAs, they can be divided into the following six categories: aconitines, lycoctonines, pyro-type, mactone-type, 7,17-seco-type, and rearranged-type. C_18_-DAs are obtained by the loss of the 18th carbon atom from C_19_-DAs. C_18_-DAs can be classified based on the presence or absence of an oxygen-containing group at the C-7 position: lappaconines and ranaconines. Based on the different groups attached to the C-4 and C-7 positions, the aconine types can be further divided into the aconosine and the lappaconine types, whereas the ranaconine types can be further classified into the leuconine and ranaconitine types [23].

**Figure 5:**
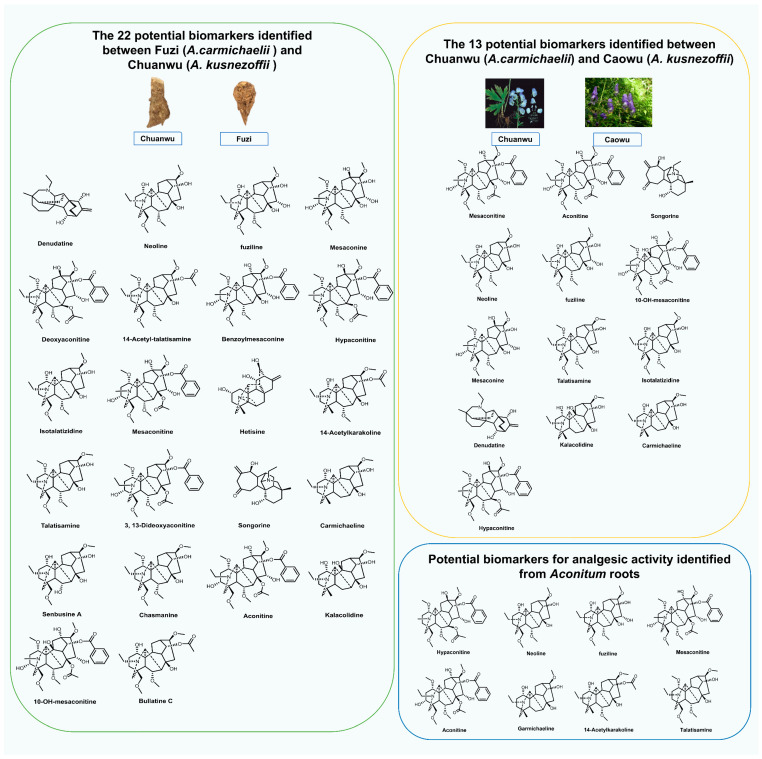
Summary of key metabolites in different tissues, species, and functions of *Aconitum* plants; source: [80,81,84].

Metabolomics has emerged as a commonly utilized method in plant research [79], and the metabolite analysis of *Aconitum* is of great importance (Table 3). With the development of metabolomics, techniques such as ultra-performance liquid chromatography quadrupole time-of-flight high-definition mass spectrometer (UPLC-Q-TOF-HDMS), ultra-high-performance liquid chromatography linear trap quadrupole orbitrap mass spectrometer (UHPLC-LTQ-Orbitrap-MS), high-performance liquid chromatography atmospheric pressure chemical ionization mass spectrometry (HPLC-APCI-MS) and ultra-high-performance liquid chromatography–diode array detector–quadrupole time-of-flight mass spectrometer–ion mobility spectrometry (UHPLC-DAD-QTOF-IMS) have been applied to the identification of alkaloid components and structures in *Aconitum* plants [80,81,82,83]. Eight secondary metabolites, including 14-acetylkarakoline, aconitine, carmichaeline, fuziline, hypaconitine, mesaconitine, neoline, and talatisamine, can be used to distinguish substitutes in the market due to the different metabolites accumulated in different *Aconitum* species [84]. There are also significant differences in the metabolites accumulated by *A. pendulum* growing at different altitudes, verifying the impact of the environment on plant secondary metabolism [85]. An analysis of various tissues of seven *Aconitum* species revealed significant differences in the accumulation of alkaloids with different carbon numbers among different species [86]. The roots of *Aconitum* are typically used as medicinal parts, while the other parts are often discarded as non-medicinal. However, through metabolomics research on different parts of *A. carmichaelii*, it has been discovered that the stems and leaves also contain aporphine alkaloids with potential medicinal effects and low toxicity, indicating their potential for further development [87]. Additionally, as a representative of toxic herbs, the detoxification process of *Aconitum* medicinal plants was dynamically analyzed by a metabolomics method, and the differences in metabolic characteristics between crude extracts and processed preparations of *Aconitum* medicinal plant and the changes in metabolite biomarkers of interest have been one of the research hotspots [88,89,90,91,92,93]. Therefore, metabolomics studies of *Aconitum* herbs are of great significance for evaluating and comparing the metabolites of different herbal tissues, identifying key metabolites or biomarkers related to the efficacy and toxicity of traditional Chinese medicine, and laying a foundation for the further identification of differential metabolites (Figure 5).

## 5. Proteomics Research in *Aconitum*

**Figure 6:**
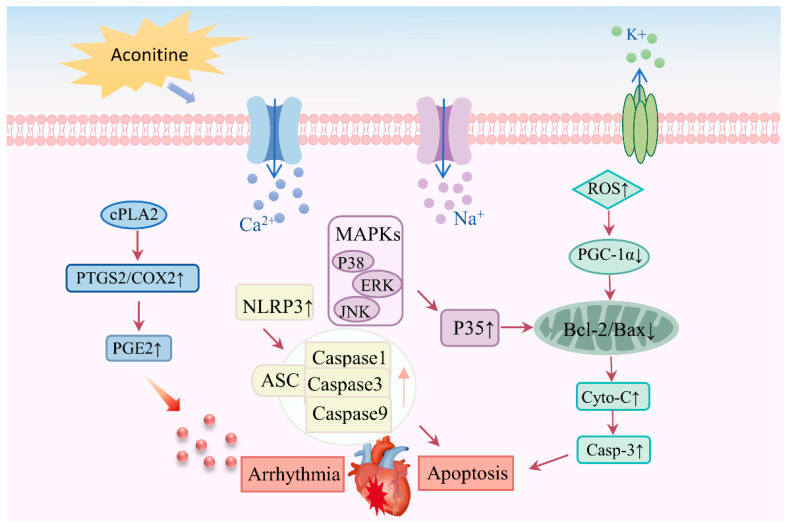
The mechanism of cardiotoxicity induced by aconitine alkaloids.

Proteomics is a high-throughput technology that allows for the analysis of proteins in cells, tissues, or organisms under specific temporal and environmental conditions [94]. Due to post-translational modifications, translocation events, and other factors, mRNA transcript abundance does not always represent the level of its cognate protein. Proteins, as the ultimate gene products, can provide more comprehensive information [95]. It is imperative to utilize this technology in order to discover disease markers and determine therapeutic targets for diseases [96]. Currently, proteomics research on *Aconitum* medicinal plants mainly focuses on the study of their active component targets and the mechanism of action in treating diseases. Various physiological effects are associated with aconitine, an active constituent of *Aconitum* [97]. However, it is highly toxic to the heart. Studies have shown that the alkaloids derived from *Aconitum* can adversely affect the functionality, morphological structure, and respiratory enzymes of cardiomyocytes. Importantly, aconitine can induce intracellular calcium overload, thereby enhancing reactive oxygen species (ROS) expression and activating the MAPK pathway, as well as regulating the levels of related proteins such as BCL2-associated X protein (Bax) and Bcl-2 to reduce the survival rate of these cells [98]. It is still unknown what the specific target of aconitine-induced cardiotoxicity is. Thereby, cytosolic phospholipase A2 (cPLA2) was discovered to be the direct target of aconitine by the application of a new proteomics technique based on “drug affinity reaction target stability” in conjunction with liquid chromatography–tandem mass spectrometry (LC-MS/MS) (Figure 6) [99]. In addition, proteomics research on *Aconitum* plants has predominantly centered on investigating the mechanism of action of their extracts or processed products in animals, utilizing tandem mass tag (TMT) protein detection technology [100,101]. However, when it comes to proteomics research on the plants themselves, the focus has been notably limited, highlighting the urgent need for more extensive studies on the plant proteome. Especially, reports on proteomics research regarding the resistance of Aconitum plants to biotic and abiotic stresses are still limited. Using mass spectrometry (MS)-based proteomics tools to study the response of plants to biotic stress remains a research field with great growth potential because it plays an important role in revealing plant–pathogen interactions, plant immune responses, signaling pathway exploration, and the discovery of natural products [95,102], suggesting an urgent need for further research on the proteomics of *Aconitum* plants.

## 6. Microbiomics Research in *Aconitum*

Microbiomics is the study of the interactions between microorganisms and their environment or host, regulating the growth, metabolism, and other activities of microbial communities [103]. Plants and endophytes form symbiotic relationships that cannot be separated, and the growth of plants depends on endophytic bacteria [104]. Their benefits include enhancing plant growth, improving stress resistance, and assisting plants to synthesize and accumulate secondary metabolites without negative effects [105]. Therefore, understanding the link between endophytic bacteria and the active components of medicinal plant hosts is critical for determining the mechanism of interaction between medicinal plant hosts and endophytic bacteria. A 16S rDNA V4 region was analyzed in the stems, roots, and leaves of endophytic bacteria of *A. carmichaelii* from multiple regions in China using high-throughput sequencing, which showed that *Aconitum* from diverse regions exhibit different levels of abundance and diversity of endophytic bacteria, indicating the correlation between microbial flora and the environment. Interestingly, the endophytic bacterial genera share a great deal of similarity with each other, as does the composition of the core microbial flora. The dominant bacterial phyla are *Proteobacteria* and *Bacteroidetes*, indicating a high degree of association between endophytic bacteria and their hosts. Among them, there is a positive correlation between the genera *Herbaspirillum* and *Massilia* and the total alkaloids in *Aconitum* [106]. In addition, high-throughput sequencing of 16S rRNA amplicons was performed on the roots of *A. carmichaelii* to analyze the bacterial community of the endophytes. The dominant bacterial phyla also included *Firmicutes* and *Actinobacteria*. Temperature and the composition of organic matter were found to be the primary factors contributing to the composition of the endophytic bacterial population. Specifically, the amount of *Lactobacillus* was positively associated with benzoylmesaconitine and benzoylaconine content. It has been suggested that endophytic *Lactobacillus* species from *A. carmichaelii* may be capable of converting the highly poisonous diester DAs into less toxic but therapeutically effective monoester DAs [107]. Twelve species of endophytic fungi were identified from the leaves, stems, and roots of the plant [108]. Studying the microbiome of *Aconitum* can help with understanding the important endophytic bacterial communities of *Aconitum* plants and the composition of endophytic bacterial communities that can produce bioactive substances, providing important evidence for guiding the further isolation of functional strains [109].

## 7. Integration Research of Multi-Omics in *Aconitum*

Plant growth and development are influenced by various factors, such as genetic information and the environment [110]. The integration of multi-omics can help us comprehensively understand the complex physiological processes and potential regulatory mechanisms involved in plant growth and development [111]. There is an interaction between plant metabolites and plant microbiota. For instance, *A. vilmorinianum* roots were collected from two locations in China, and analyses of alkaloid metabolism were conducted using high-throughput sequencing and ultra-high-performance liquid chromatography–tandem mass spectrometry (UHPLC-MS/MS), along with microbiomics profiling of bacterial and fungal microbiome. Significant differences were found in the alkaloid metabolites enriched in samples from different regions and the recruited microbiota. Additionally, 137 bacterial and 17 fungal species were found to be associated with different metabolites, indicating that these microorganisms may affect alkaloid biosynthesis in the host plant. The results contribute to further research on the interaction between plants, rhizosphere microorganisms, and the impact of microorganisms on plant metabolites, thereby promoting the accumulation of secondary metabolites [112].

The production of plant secondary metabolites involves a complicated regulatory network [113], and the *Aconitum* plants have been studied for their secondary metabolite biosynthesis pathways. The combined analysis of transcriptomics and metabolomics could help to systematically elucidate the regulatory mechanisms of plant secondary metabolite synthesis [114]. The biosynthesis pathway of C_20_-diterpene alkaloids from *A. gymnandrum* was studied by combining transcriptome and metabolome data. In this study, methyl jasmonate (MJ) was used to induce *A. gymnandrum* in the bacteria-free vaccine, transcriptome changes were analyzed by single-molecule real-time sequencing (SMRT) and Illumina sequencing, and the metabolite induction effect was evaluated by ultra-performance liquid chromatography–quadrupole time-of-flight mass spectrometry (UPLC-Q-TOF-MS) technique. The integration of these approaches provides insights into the biosynthetic pathways of DAs in *Aconitum* species [55]. Another study on *A. vilmorinianum* combined genome and transcriptome data to identify genes encoding KOX enzymes, which promote the activation of diterpenoid compounds, and ATF, responsible for inserting nitrogen atoms into the diterpenoid skeleton. Additionally, it was found that genes encoding DAs are abundant in leaves, suggesting that multiple DAs are produced in the roots from a precursor synthesized in the aerial parts. By combining metabolomics and transcriptomics, a gene–metabolite regulatory network was established, and 12 hub genes were found to be correlated with the content of multiple aconitine alkaloids, promoting an understanding of DA biosynthesis and origin evolution in *Aconitum* plants. Integrating genome sequencing, metabolomics, transcriptomics, proteomics, and other multi-omics approaches will accelerate the identification of biosynthetic genes and biochemical pathways for secondary metabolite biosynthesis.

## 8. Conclusions and Perspectives

There is an increasing interest in studying the biological processes of *Aconitum* medicinal plants due to their high medicinal and economic values. As a consequence, omics methods have emerged as efficient methods for obtaining a wealth of information on *Aconitum* genome sequences, transcription patterns, protein expression patterns, metabolites, plant endo microbiota, and more. Omics research on *Aconitum* medicinal plants has made many breakthroughs over the past few years. Particularly noteworthy is the recent report on the genome of *A. vilmorinianum* at the chromosomal level, which represents a significant breakthrough in the molecular biology of *Aconitum* plants. Currently, the main obstacle in the *Aconitum* omics files is the lack of high-quality assembly. In the last decades, herb genomics has seen remarkable progress with the successful creation of high-quality assemblies for medicinal plants, including both telomere-to-telomere and phase-resolved assemblies [115,116,117]. We would expect more high-quality *Aconitum* genome assemblies in the near future, which could lay the groundwork for other omics works, such as transcriptomics and proteomics. Additionally, molecular breeding and the accumulation of key active ingredients will be facilitated by the availability of high-quality *Aconitum* genomes. Omics analysis provides important information for a deeper, more comprehensive, and systematic understanding of *Aconitum*, offering broader possibilities for future germplasm resource innovation and quality improvement. However, there are still some limitations in the research process, and the future development of *Aconitum* omics would mainly focus on the following aspects.

Despite the identification of many genes responsible for the DA biosynthesis pathway of major medicinal plants in the *Aconitum* genus, the functions of most of these genes remain uncertain. In addition, *Aconitum* plants generally live in arid environments with harsh conditions, but our understanding of their molecular mechanisms of drought resistance is limited. The key genes that regulate growth and stress resistance in *Aconitum* should be located in the genome and functionally elucidated through in vitro and in vivo enzyme experiments.

The field of metabolomics research on *Aconitum* plants is extensive and plays a crucial foundation in the identification of plant chemical constituents, species discrimination, and investigations of changes in constituents after processing. Traditional Chinese medicine scientifically guides the formation and quality of *Aconitum* medicinal materials. Next, we need to address the unresolved part of biochemical pathways and metabolic networks and further explore the relationships between phenotypes and *Aconitum*-specific or species-specific metabolites.

Limited research has been conducted on the proteomics of *Aconitum* plants, emphasizing the necessity of enhancing the investigation of *Aconitum* proteomics, specifically an in-depth analysis of how it is affected by varying developmental stages and stress conditions.

At present, the study of *Aconitum* plant microbiomes is in its initial phases, with a focus on a few model species when researching the endophytes of *Aconitum*. The improvement of active substance production in *Aconitum* requires a stronger focus on researching the structure, function, and physiology of its related microbiome, as well as the isolation of functional strains.

Our goal is to identify the genes and primary metabolites that impact metabolism regulation in *Aconitum* medicinal plants. Ultimately, we would aim to use molecular genetic breeding techniques to develop superior varieties with increased metabolite levels for production. In order to achieve this goal, we should accelerate the integration of a multitude of omics so as to acquire a deeper understanding of the intricate biosynthetic regulatory networks of *Aconitum* and the intricate mechanisms of interaction between root microorganisms.

Plants of the genus *Aconitum* are highly valuable ornamental flowers with unique blue-purple hues. Currently, there is limited research on genes associated with the traits of *Aconitum* plants. By utilizing genomic and transcriptomic data mining, we can identify genes related to unique flower shapes, vivid colors, and strong cold resistance, providing a molecular basis for the subsequent cultivation of excellent ornamental flower varieties.

## Figures and Tables

**Table 1:** Chromosome number of important *Aconitum* species.

2n = 12	2n = 16 (2×)	2n = 32 (4×)	2n = 48 (6×)
*A. fletcheranum* G.Taylor	*A. novoluridum* Munz *A. scaposum* Franch *A. alboviolaceum* Kom. *A. finetianum* Hand.-Mazz. *A. longecassidatum* Nakai *A. leucostomum* Vorosch. *A. Leucostomum* var. *hopeiense* W. T. Wang *A. wangyedianense* Y. Z. Zhao *A. shennongjiaense* Q. Gao & Q. E.Yang *A. sinomontanum* Nakai *A. umbrosum* (Korsh.) Kom. *A. barbatum* Pers. var. *barbatum* *A. barbatum* var. *puberulum* Ledeb.	*A. brevicalcaratum* (Finet & Gagnep.) var. *brevicalcaratum* *A. brevicalcaratum* var. *parviflorum* Chen & Liu *A. chrysotrichum* W. T. Wang *A. crassiflorum* Hand.-Mazz. *A. angustius* W. T. Wang	*A. apetalum* (Huth) B. Fedtsch. ex Steinb.

Sources: [32].

**Table 2:** Summary of *Aconitum* chloroplast genomes.

Species	Genome Size (bp)	Total Number of Genes	GC Content	LSC Length (bp)	SSC Length (bp)	A Pair of IR Regions Length (bp)	References
*A. angustius*	156,109	126	38.0%	86,719	16,914	26,225	[38]
*A. austroyunnanense*	155,818	131	38.1%	86,555	17,007	26,128	[39]
*A. brachypodum*	155,651	132	38.0%	86,292	16,933	26,213	[40]
*A. carmichaelii*	155,737	129	38.1%	86,330	17,021	26,193	[41]
*A. coreanum*	157,024	132	38.0%	87,637	16,901	26,243	[42]
*A. contortum*	155,653	132	38.1%	86,267	16,944	26,221	[43]
*A. delavayi*	155,733	132	38.1%	86,362	16,913	26,229	[44]
*A. episcopale*	155,853	129	38.1%	86,443	16,938	26,236	[45]
*A. favum*	155,654	129	38.1%	86,390	16,968	26,148	[46]
*A. pendulum*	155,597	131	38.1%	86,336	16,961	26,150	[47]
*A. piepunense*	155,836	130	38.1%	86,433	16,945	25,927	[48]
*A. puchonroenicum*	155,631	111	38.05%	86,689	17,088	25,927	[49]
*A. tschangbaischanense*	155,881	129	38.1%	86,330	16,914	26,225	[35]
*A. transsectum*	155,872	131	38.07%	87,671	18,891	25,894	[50]
*A. tanguticum*	157,114	112	38.0%	87,559	17,045	26,255	[51]

**Table 3:** A summary of metabolomic studies in *Aconitum*.

Experimental Material	Tissue	Aim/Result	Method	References
*A. carmichaelii* and *A. kusnezoffii*	Roots	Studied constituents in the root of two kinds of *Aconitum* species	UPLC-Q-TOF-HDMS	[80]
*A. heterophyllum*	Flowers, leaves stems, roots, and tubers	Development of methods for the characterization and quantitative analysis of alkaloid compounds	UHPLC-DAD-QTOF-IMS	[81]
*A. carmichaelii*	Roots	A quick characterization and detection process to detect diester-DAs (DDAs)	UHPLC-LTQ-Orbitrap-MS	[82]
*A. yesoense* var. *Macroyesoense* *A. japonicum*	Roots	Separation of stereoisomeric aconitine alkaloids with different configurations	HPLC-APCI-MS	[83]
*A. carmichaelii*, *A. bulleyanum*, *A. iochanicum*, *A. ouvrardianum*, *A. fengii* *A. transsectum*, *A. pukeense*, *A. weixiense*	Roots	The difference between genuine medicinal plants and common adulterations	UHPLC-Q-TOF/MS	[84]
*A. pendulum*	Rhizomes	Environmental differences affect the accumulation of secondary metabolites in *A. pendulum*	UPLC-MS/MS	[85]
*A. carmichaelii*, *A. stylosum* *A. Pendulum*, *A. tanguticum* *A. sinomontanum* *A. vilmorinianum* *A. gym-nandrum*	Principal roots, lateral roots, leaf apexes, leaves, flowers xylem, stems	Distribution and main metabolites of DAs in *Aconitum*	UHPLC-Q-TOF/MS	[86]
*A. carmichaelii*	Principal roots, lateral roots, stems and leaves	Different metabolites in different parts of *Aconitum* were associated with toxicity to screen toxicity markers	UHPLC-Q-orbitrap MS/MS	[87]
*A. carmichaelii*	Roots	Seventeen biomarkers were associated with *A. carmichaelii* toxicity, and the expression levels of most of them were effectively regulated to normal levels by the combination of drugs	UPLC-Q-TOF/MS	[88]
*A. carmichaelii* (Fuzi)	Lateral roots	There were differences in chemical composition between raw and processed Fuzi for different steaming times; 4.0 h is the proper time for toxicity attenuation and efficacy reservation. A total of 42 metabolic markers were identified to discriminate raw Fuzi and those steamed for 4.0 and 8.0 h	DESI-MSI and HPLC	[89]
*A. carmichaelii* (Fuzi)	Lateral roots	Fuzi and its processed preparations differ in their metabolic profiles, and significant changes in nineteen metabolite biomarkers were detected in Fuzi samples, as well as in three preparations	UPLC-Q-TOF-HDMS	[90]
*A. carmichaelii* (Fuzi)	Lateral roots	Fuzi and its processed preparations differ in their metabolic profiles, and significant changes in nineteen metabolite biomarkers were detected in Fuzi samples, as well as in three preparations	UPLC-Q-TOF-HDMS	[90]
*A. carmichaelii* (Fuzi)	Lateral roots	A quantification method for the simultaneous determination of 15 key components to compare different processed products of Fuzi; the processing procedures were optimized to obtain new processed products of Heishunpian (black slices) with less toxicity	UPLC-Q-TOF/MS and UHPLC-QqQ-MS/MS	[91]
*A. pendulum* (Tiebangchui)	Dried roots	Tiebangchui raw and their processed products were investigated for their chemical composition, transformation mechanism, and spatial distribution of metabolites	UPLC-Q-TOF/MS and DESI-MSI	[92]
*A. pendulum*	Dried roots	The visual analysis of 18 chemical changes in raw *A. pendulum* and its liquid-cooked and sand-fried products was established	UPLC-Q-TOF/MS and HPTLC/PAD-DESI-MSI	[93]

## Data Availability

Dataset available on request from the authors.

## References

[1] Luo Y., Zhang F., Yang Q.E. (2005). Phylogeny of *Aconitum* Subgenus *Aconitum* (Ranunculaceae) Inferred from ITS Sequences. Plant Syst. Evol..

[2] Shyaula S.L. (2012). Phytochemicals, Traditional Uses and Processing of *Aconitum* Species in Nepal. Nepal J. Sci. Technol..

[3] Tynkevich Y.O., Novikov A.V., Chorney I.I., Volkov R.A. (2022). Organization of the 5S rDNA Intergenic Spacer and Its Use in the Molecular Taxonomy of the Genus *Aconitum* L.. Cytol. Genet..

[4] Jabeen N., Khan S. (2013). Distribution and Taxonomy of Genus *Aconitum* in Kashmir: Potent Medicinal Resource of Himalayan Valley. Chiang Mai J. Sci..

[5] Baldini R.M., Cota-Sánchez J.H., Aedo C. (2021). Is the Demise of Plant Taxonomy in Sight? Maybe Yes, Maybe No. Webbia.

[6] Tatsuzawa F., Mukai C., Igarashi M., Hishida A., Satta N., Honda K., Nakajo S., Takehara A., Tanikawa N. (2019). Anthocyanins and Anthocyanidins in the Flowers of *Aconitum* (Ranunculaceae). Biochem. Syst. Ecol..

[7] Fu Y.P., Zou Y.F., Lei F.Y., Wangensteen H., Inngjerdingen K.T. (2022). *Aconitum carmichaelii* Debeaux: A Systematic Review on Traditional Use, and the Chemical Structures and Pharmacological Properties of Polysaccharides and Phenolic Compounds in the Roots. J. Ethnopharmacol..

[8] Guo Q., Xia H., Meng X., Shi G., Xu C., Zhu C., Zhang T., Shi J. (2018). C19-Diterpenoid Alkaloid Arabinosides from an Aqueous Extract of the Lateral Root of *Aconitum carmichaelii* and Their Analgesic Activities. Acta Pharm. Sin. B.

[9] Li C.Y., Zhou Z., Xu T., Wang N.Y., Tang C., Tan X.Y., Feng Z.G., Zhang Y., Liu Y. (2022). *Aconitum pendulum* and *Aconitum flavum*: A Narrative Review on Traditional Uses, Phytochemistry, Bioactivities, and Processing Methods. J. Ethnopharmacol..

[10] Yin T., Zhou H., Cai L., Ding Z. (2019). Non-Alkaloidal Constituents from the Genus *Aconitum*: A Review. RSC Adv..

[11] Nyirimigabo E., Xu Y., Li Y., Wang Y., Agyemang K., Zhang Y. (2015). A Review on Phytochemistry, Pharmacology and Toxicology Studies of *Aconitum*. J. Pharm. Pharmacol..

[12] He G., Wang X., Liu W., Li Y., Shao Y., Liu W., Liang X., Bao X. (2023). Chemical Constituents, Pharmacological Effects, Toxicology, Processing and Compatibility of Fuzi (Lateral Root of *Aconitum carmichaelii* Debx): A Review. J. Ethnopharmacol..

[13] Tiwari S., Acharya P., Solanki B., Sharma A.K., Rawat S. (2023). A Review on Efforts for Improvement in Medicinally Important Chemical Constituents in *Aconitum* through Biotechnological Interventions. 3 Biotech.

[14] Chan T.Y.K. (2009). Aconite Poisoning. Clin. Toxicol..

[15] Tao H., Liu X., Tian R., Liu Y., Zeng Y., Meng X., Zhang Y. (2023). A Review: Pharmacokinetics and Pharmacology of Aminoalcohol-Diterpenoid Alkaloids from *Aconitum* Species. J. Ethnopharmacol..

[16] Liu S., Li F., Li Y., Li W., Xu J., Du H. (2017). A Review of Traditional and Current Methods Used to Potentially Reduce Toxicity of *Aconitum* Roots in Traditional Chinese Medicine. J. Ethnopharmacol..

[17] Shen X., Yin T., Li X., Ma J., Lv Q., Zhang G. (2020). Diterpenoid Alkaloids with Chemotaxonomic Significance from *Aconitum spathulatum*. Biochem. Syst. Ecol..

[18] Qiu Z.D., Chen J.L., Zeng W., Ma Y., Chen T., Tang J.F., Lai C.J.S., Huang L.Q. (2020). Real-Time Toxicity Prediction of *Aconitum* Stewing System Using Extractive Electrospray Ionization Mass Spectrometry. Acta Pharm. Sin. B.

[19] Qiu Z.D., Zhang X., Wei X.Y., Chingin K., Xu J.Q., Gao W., Yang B., Wang S.L., Tan T., Liu E.H. (2021). Online Discovery of the Molecular Mechanism for Directionally Detoxification of Fuzi Using Real-Time Extractive Electrospray Ionization Mass Spectrometry. J. Ethnopharmacol..

[20] Tian M., Jin B., Chen L., Ma R., Ma Q., Li X., Chen T., Guo J., Ge H., Zhao X. (2023). Functional Diversity of Diterpene Synthases in *Aconitum* Plants. Plant Physiol. Biochem..

[21] Wang Y., Shi L., Song F., Liu Z., Liu S. (2003). Exploring the Ester-exchange Reactions of Diester-diterpenoid Alkaloids in the Aconite Decoction Process by Electrospray Ionization Tandem Mass Spectrometry. Rapid Commun. Mass Spectrometr..

[22] Wang Y., Liu Z., Song F., Liu S. (2003). Study on *Aconitum* diterpenoid alkaloids from flowers of *Aconitum kusnezoffii* and its decoction by ESI-MS. Yao Xue Xue Bao.

[23] Shen Y., Liang W.J., Shi Y.N., Kennelly E.J., Zhao D.K. (2020). Structural Diversity, Bioactivities, and Biosynthesis of Natural Diterpenoid Alkaloids. Nat. Prod. Rep..

[24] Zhan C., Shen S., Yang C., Liu Z., Fernie A.R., Graham I.A., Luo J. (2022). Plant Metabolic Gene Clusters in the Multi-Omics Era. Trends Plant Sci..

[25] Smith Olsen C., Overgaard Larsen H. (2003). Alpine Medicinal Plant Trade and Himalayan Mountain Livelihood Strategies. Geogr. J..

[26] Kakkar R.A., Haneen M.A., Parida A.C., Sharma G. (2023). The Known, Unknown, and the Intriguing about members of a Critically Endangered Traditional Medicinal Plant Genus *Aconitum*. Front. Plant Sci..

[27] Weldemichael M.Y., Gebremedhn H.M. (2023). Omics Technologies towards Sesame Improvement: A Review. Mol. Biol. Rep..

[28] Wang Y., Tong Y., Adejobi O.I., Wang Y., Liu A. (2022). Research Advances in Multi-Omics on the Traditional Chinese Herb *Dendrobium officinale*. Front. Plant Sci..

[29] Medema M.H., Osbourn A. (2016). Computational Genomic Identification and Functional Reconstitution of Plant Natural Product Biosynthetic Pathways. Nat. Prod. Rep..

[30] Lavania U.C. (2020). Plant Speciation and Polyploidy: In Habitat Divergence and Environmental Perspective. Nucleus.

[31] Joachimiak A.J., Hasterok R., Sliwinska E., Musiał K., Grabowska-Joachimiak A. (2018). FISH-Aimed Karyotype Analysis in *Aconitum* Subgen. *Aconitum* Reveals Excessive rDNA Sites in Tetraploid Taxa. Protoplasma.

[32] Hong Y., Gao Q., Luo Y., Luo J., Zhang Y., Yuan Q., Yang Q. (2016). Karyology of *Aconitum* Subgenus *Lycoctonum* (Ranunculaceae) from China, with a Report of the New Base Chromosome Number x = 6 in the Genus *Aconitum*. Nord. J. Bot..

[33] Gao Q., Ren C., Yang Q. (2012). Taxonomic Status and Distributional Range of *Aconitum angustius* (Ranunculaceae) Based on Cytological Evidence. Nord. J. Bot..

[34] Zhao D., Zhang Y., Ren H., Shi Y., Dong D., Li Z., Cui G., Shen Y., Mou Z., Kennelly E.J. (2023). Multi-omics Analysis Reveals the Evolutionary Origin of Diterpenoid Alkaloid Biosynthesis Pathways in *Aconitum*. JIPB.

[35] Liu X., Zhu J., Jiang M., Guan S., Zhang L., Zhao H. (2023). The Complete Chloroplast Genome Sequence of *Aconitum tschangbaischanense* (Ranunculaceae). Mitochondrial DNA Part B.

[36] Daniell H., Jin S., Zhu X., Gitzendanner M.A., Soltis D.E., Soltis P.S. (2021). Green Giant—A Tiny Chloroplast Genome with Mighty Power to Produce High-value Proteins: History and Phylogeny. Plant Biotechnol. J..

[37] Xia C., Wang M., Guan Y., Li J. (2022). Comparative Analysis of the Chloroplast Genome for *Aconitum* Species: Genome Structure and Phylogenetic Relationships. Front. Genet..

[38] Kong H., Liu W., Yao G., Gong W. (2017). A Comparison of Chloroplast Genome Sequences in *Aconitum* (Ranunculaceae): A Traditional Herbal Medicinal Genus. PeerJ.

[39] Cheng Z.D., He J., Zhang Y.M., Yang C.W., Ma X.X., Li G.D. (2020). The Complete Chloroplast Genome Sequence of *Aconitum austroyunnanense* W. T. Wang (Ranunculaceae): A Medicinal Plant Endemic to China. Mitochondrial DNA Part B.

[40] Meng J., Zhang L., Li X., He J. (2019). The Complete Plastid Genome Sequence of *Aconitum brachypodum* (Ranunculaceae): An Endangered Species Endemic to China. Mitochondrial DNA Part B.

[41] Yang J., Zeng X., Guo S. (2018). Characterization of the Complete Chloroplast Genome of the Perennial Herb *Aconitum carmichaelii* (Ranunculales: Ranunculaceae). Conserv. Genet. Resour..

[42] Kim Y., Yi J.S., Min J., Xi H., Kim D.Y., Son J., Park J., Jeon J.I. (2019). The Complete Chloroplast Genome of *Aconitum coreanum* (H. Lév.) Rapaics (Ranunculaceae). Mitochondrial DNA Part B.

[43] Meng J., Li X., Li H., Yang J., Wang H., He J. (2018). Comparative Analysis of the Complete Chloroplast Genomes of Four *Aconitum* Medicinal Species. Molecules.

[44] Yu W.B., Wang H., Liu M.L., Grabovskaya-Borodina A.E., Li D.Z. (2018). Phylogenetic Approaches Resolve Taxonomical Confusion in Pedicularis (Orobanchaceae): Reinstatement of *Pedicularis Delavayi* and Discovering a New Species *Pedicularis milliana*. PLoS ONE.

[45] Xia C., Wang M., Guan Y., Li Y., Li J. (2022). Comparative Analysis of Complete Chloroplast Genome of Ethnodrug *Aconitum* Episcopale and Insight into Its Phylogenetic Relationships. Sci Rep..

[46] Liu Y., Yu S., You F. (2020). Characterization of the Complete Chloroplast Genome of *Aconitum Flavum* (Ranunculaceae). Mitochondrial DNA Part B.

[47] Wang Z.H., Li Y.Q. (2020). Characterization of the Complete Chloroplast Genome of *Aconitum pendulum* (Ranunculaceae), an Endemic Medicinal Herb. Mitochondrial DNA Part B.

[48] Ni X., Li J., Li Y., Zhang H., Duan B., Chen X., Xia C. (2022). The Complete Chloroplast Genome of *Aconitum piepunense* (Ranunculaceae) and Its Phylogenetic Analysis. Mitochondrial DNA Part B.

[49] Lim C.E., Ryul B.K., Lee J.D., Jung K.D., Noh T.K., Lee B.Y. (2020). The Complete Chloroplast Genome of *Aconitum puchonroenicum* Uyeki & Sakata (Ranunculaceae), a Rare Endemic Species in Korea. Mitochondrial DNA Part B.

[50] Yanfei N., Tai S., Chunhua W., Jia D., Fazhong Y. (2023). Complete Chloroplast Genome Sequences of the Medicinal Plant *Aconitum transsectum* (Ranunculaceae): Comparative Analysis and Phylogenetic Relationships. BMC Genom..

[51] Li Q., Li X., Qieyang R., Nima C., Dongzhi D., Duojie, Guo X. (2020). Characterization of the Complete Chloroplast Genome of the Tangut Monkshood *Aconitum tanguticum* (Ranunculales: Ranunculaceae). Mitochondrial DNA Part B.

[52] Liu J., Gao Y., Zhang X., Hao Z., Zhang H., Gui R., Liu F., Tong C., Wang X. (2024). Transcriptome Sequencing Analysis of Bovine Mammary Epithelial Cells Induced by Lipopolysaccharide. Anim. Biotechnol..

[53] Rai M., Rai A., Kawano N., Yoshimatsu K., Takahashi H., Suzuki H., Kawahara N., Saito K., Yamazaki M. (2017). De Novo RNA Sequencing and Expression Analysis of *Aconitum carmichaelii* to Analyze Key Genes Involved in the Biosynthesis of Diterpene Alkaloids. Molecules.

[54] Pal T., Malhotra N., Chanumolu S.K., Chauhan R.S. (2015). Next-Generation Sequencing (NGS) Transcriptomes Reveal Association of Multiple Genes and Pathways Contributing to Secondary Metabolites Accumulation in Tuberous Roots of *Aconitum heterophyllum* Wall. Planta.

[55] Chen L., Tian M., Jin B., Yin B., Chen T., Guo J., Tang J., Cui G., Huang L. (2022). Integrating Metabolomics and Transcriptomics to Unveil Atisine Biosynthesis in *Aconitum gymnandrum* Maxim. Int. J. Mol. Sci..

[56] Li Y.G., Mou F.J., Li K.Z. (2021). De Novo RNA Sequencing and Analysis Reveal the Putative Genes Involved in Diterpenoid Biosynthesis in *Aconitum vilmorinianum* Roots. 3 Biotech.

[57] Tian M., Chen L., Cui G. (2021). Transcriptome analysis to identify genes involved in the biosynthesis of aconitines in *Aconitum pendulum*. Acta Pharm. Sin..

[58] Zi J., Mafu S., Peters R.J. (2014). To Gibberellins and Beyond! Surveying the Evolution of Diterpenoid Metabolism. Annu. Rev. Plant Biol..

[59] Mendoza-Poudereux I., Kutzner E., Huber C., Segura J., Arrillaga I., Eisenreich W. (2017). Dynamics of Monoterpene Formation in Spike Lavender Plants. Metabolites.

[60] Zhao D., Shen Y., Shi Y., Shi X., Qiao Q., Zi S., Zhao E., Yu D., Kennelly E.J. (2018). Probing the Transcriptome of *Aconitum carmichaelii* Reveals the Candidate Genes Associated with the Biosynthesis of the Toxic Aconitine-Type C19-Diterpenoid Alkaloids. Phytochemistry.

[61] Zhao P.J., Gao S., Fan L.M., Nie J.L., He H.P., Zeng Y., Shen Y.M., Hao X.J. (2009). Approach to the Biosynthesis of Atisine-Type Diterpenoid Alkaloids. J. Nat. Prod..

[62] Kumar V., Malhotra N., Pal T., Chauhan R.S. (2016). Molecular Dissection of Pathway Components Unravel Atisine Biosynthesis in a Non-Toxic *Aconitum* Species, *A. heterophyllum* Wall. 3 Biotech.

[63] Zhang K., Wang N., Gao X., Ma Q. (2022). Integrated Metabolite Profiling and Transcriptome Analysis Reveals Tissue-Specific Regulation of Terpenoid Biosynthesis in *Artemisia argyi*. Genomics.

[64] Liao H., Quan H., Huang B., Ji H., Zhang T., Chen J., Zhou J. (2023). Integrated Transcriptomic and Metabolomic Analysis Reveals the Molecular Basis of Tissue-Specific Accumulation of Bioactive Steroidal Alkaloids in *Fritillaria unibracteata*. Phytochemistry.

[65] Feng M., Chen C., Qu-Bie J., Qu-Bie A., Bao X., Cui Q., Yan X., Li Y., Liu Y., Zhang S. (2022). Metabolome and Transcriptome Associated Analysis of Sesquiterpenoid Metabolism in *Nardostachys jatamansi*. Front. Plant Sci..

[66] Wang X., Hu H., Wu Z., Fan H., Wang G., Chai T., Wang H. (2021). Tissue-Specific Transcriptome Analyses Reveal Candidate Genes for Stilbene, Flavonoid and Anthraquinone Biosynthesis in the Medicinal Plant *Polygonum cuspidatum*. BMC Genom..

[67] Malhotra N., Sood H., Chauhan R.S. (2016). Transcriptome-Wide Mining Suggests Conglomerate of Genes Associated with Tuberous Root Growth and Development in *Aconitum heterophyllum* Wall. 3 Biotech.

[68] Tang M., Zhao W., Xing M., Zhao J., Jiang Z., You J., Ni B., Ni Y., Liu C., Li J. (2021). Resource Allocation Strategies among Vegetative Growth, Sexual Reproduction, Asexual Reproduction and Defense during Growing Season of *Aconitum kusnezoffii* Reichb. Plant J..

[69] Bai S., Sartagnuud S., Wang T., Bao G., Bao S., Ao W. (2022). De Nove Transcriptome Sequencing Identifies Genes Involved in Aconitine-Type Alkaloids Biosynthesis in *Aconitum kusnezoffii* Reichb. Pharmacol. Res.—Mod. Chin. Med..

[70] Ma L., Wang X.N. (2015). Cloning and Expression Pattern Analysis of Av-F3’5’H Gene from *Aconitum vilmorinianum*. Southwest China J. Agric. Sci..

[71] Mitu S.A., Cummins S.F., Reddell P.W., Ogbourne S.M. (2020). Transcriptome Analysis of the Medicinally Significant Plant *Fontainea picrosperma* (Euphorbiaceae) Reveals Conserved Biosynthetic Pathways. Fitoterapia.

[72] Sun B., Wang P., Guan M., Jia E., Li Q., Li J., Zhou Z., Ma P. (2023). Tissue-Specific Transcriptome and Metabolome Analyses Reveal Candidate Genes for Lignan Biosynthesis in the Medicinal Plant *Schisandra sphenanthera*. BMC Genom..

[73] Mao L., Jin B., Chen L., Tian M., Ma R., Yin B., Zhang H., Guo J., Tang J., Chen T. (2021). Functional Identification of the Terpene Synthase Family Involved in Diterpenoid Alkaloids Biosynthesis in *Aconitum carmichaelii*. Acta Pharm. Sin. B.

[74] Moghe G., Kruse L.H., Petersen M., Scossa F., Fernie A.R., Gaquerel E., D’Auria J.C. (2023). BAHD Company: The Ever-Expanding Roles of the BAHD Acyltransferase Gene Family in Plants. Annu. Rev. Plant Biol..

[75] Bayer A., Ma X., Stöckigt J. (2004). Acetyltransfer in Natural Product Biosynthesis—Functional Cloning and Molecular Analysis of Vinorine Synthase. Bioorgan. Med. Chem..

[76] Walker K., Croteau R. (2000). Taxol Biosynthesis: Molecular Cloning of a Benzoyl- CoA: Taxane 2α-*O*-Benzoyltransferase cDNA from *Taxus* and Functional Expression in *Escherichia coli*. Proc. Natl. Acad. Sci. USA.

[77] Zhou J., Liu W., Kong H., Gong W. (2018). Identification and Characterization of Microsatellites in *Aconitum reclinatum* (Ranunculaceae), a Rare Species Endemic to North America. Appl. Plant Sci..

[78] Van Der Hooft J.J.J., Mohimani H., Bauermeister A., Dorrestein P.C., Duncan K.R., Medema M.H. (2020). Linking Genomics and Metabolomics to Chart Specialized Metabolic Diversity. Chem. Soc. Rev..

[79] Gonda S. (2020). Special Issue: Plant Metabolomics. Metabolites.

[80] Sun H., Wang M., Zhang A., Ni B., Dong H., Wang X. (2013). UPLC–Q-TOF–HDMS Analysis of Constituents in the Root of Two Kinds of *Aconitum* Using a Metabolomics Approach. Phytochem. Anal..

[81] Punia A., Joshi R., Kumar R. (2022). Identification and Quantification of Eight Alkaloids in *Aconitum heterophyllum* Using UHPLC-DAD-QTOF-IMS: A Valuable Tool for Quality Control. Phytochem. Anal..

[82] Zhang J., Huang Z.H., Qiu X.H., Yang Y.M., Zhu D.Y., Xu W. (2012). Neutral Fragment Filtering for Rapid Identification of New Diester-Diterpenoid Alkaloids in Roots of *Aconitum carmichaelii* by Ultra-High-Pressure Liquid Chromatography Coupled with Linear Ion Trap-Orbitrap Mass Spectrometry. PLoS ONE.

[83] Wada K. (2002). Studies on structural elucidation of *Aconitum* diterpenoid alkaloid by LC-APCI-MS and effects of *Aconitum* diterpenoid alkaloid on cutaneous blood flow. Yakugaku Zasshi.

[84] Zhao D., Shi Y., Zhu X., Liu L., Ji P., Long C., Shen Y., Kennelly E. (2018). Identification of Potential Biomarkers from *Aconitum carmichaelii*, a Traditional Chinese Medicine, Using a Metabolomic Approach. Planta Med..

[85] Wang J.J., Lou H.Y., Liu Y., Han H.P., Ma F.W., Pan W.D., Chen Z. (2022). Profiling Alkaloids in *Aconitum pendulum* N. Busch Collected from Different Elevations of Qinghai Province Using Widely Targeted Metabolomics. Phytochemistry.

[86] Chen L.L., Lai C.J.S., Mao L.Y., Yin B.W., Tian M., Jin B.L., Wei X.Y., Chen J.L., Ge H., Zhao X. (2021). Chemical Constituents in Different Parts of Seven Species of *Aconitum* Based on UHPLC-Q-TOF/MS. J. Pharm. Biomed. Anal..

[87] Zhou Y., Qu C., Yan H., Chu T., Wu J., Kang Q., Peng C., Wang Y., Tan Y. (2024). Unlocking the Hidden Potential: Enhancing the Utilization of Stems and Leaves through Metabolite Analysis and Toxicity Assessment of Various Parts of *Aconitum carmichaelii*. J. Ethnopharmacol..

[88] Dong H., Yan G.L., Han Y., Sun H., Zhang A.H., Li X.N., Wang X.J. (2015). UPLC-Q-TOF/MS-Based Metabolomic Studies on the Toxicity Mechanisms of Traditional Chinese Medicine Chuanwu and the Detoxification Mechanisms of Gancao, Baishao, and Ganjiang. Chin. J. Nat. Med..

[89] Liu Y., Yang X., Zhou C., Wang Z., Kuang T., Sun J., Xu B., Meng X., Zhang Y., Tang C. (2022). Unveiling Dynamic Changes of Chemical Constituents in Raw and Processed Fuzi with Different Steaming Time Points Using Desorption Electrospray Ionization Mass Spectrometry Imaging Combined with Metabolomics. Front. Pharmacol..

[90] Sun H., Ni B., Zhang A., Wang M., Dong H., Wang X. (2012). Metabolomics Study on Fuzi and Its Processed Products Using Ultra-Performance Liquid-Chromatography/Electrospray-Ionization Synapt High-Definition Mass Spectrometry Coupled with Pattern Recognition Analysis. Analyst.

[91] Yu Y., Yao C., Zhang J., Bi Q., Wei W., Li Z., Li J., Yao S., Huang Y., Qu H. (2023). Comprehensive Quality Evaluation of Aconiti Lateralis Radix Praeparata Based on Pseudotargeted Metabolomics and Simultaneous Determination of Fifteen Components, and Development of New Processed Products of Black Slices with Less Toxicity. J. Pharm. Biomed. Anal..

[92] Li C.Y., Sha M.X., Pei Z.Q., Zhou Z., Tang C., Liu Y., Zhang Y. (2023). Dynamic Variations in the Chemical Constituents of Tiebangchui Stir-Fried with Zanba by Integrating UPLC-Q-TOF-MS Based Metabolomics and DESI-MSI. Arab. J. Chem..

[93] Liu Y., Li M., Fu X., Zhang Y., Tang C. (2024). An Integrated Strategy of UPLC-Q-TOF/MS and HPTLC/PAD-DESI-MSI for the Analysis of Chemical Variations: A Case Study of Tibetan Medicine Tiebangchui. J. Pharm. Anal..

[94] (2021). Proteomics. Nat. Biotechnol..

[95] Yan S., Bhawal R., Yin Z., Thannhauser T.W., Zhang S. (2022). Recent Advances in Proteomics and Metabolomics in Plants. Mol. Hortic..

[96] Cifani P., Kentsis A. (2017). Towards Comprehensive and Quantitative Proteomics for Diagnosis and Therapy of Human Disease. Proteomics.

[97] Wani T.A., Kaloo Z.A., Dangroo N.A. (2022). *Aconitum heterophyllum* Wall. Ex Royle: A Critically Endangered Medicinal Herb with Rich Potential for Use in Medicine. J. Integr. Med..

[98] Wang Y., Shan Y., Wang Y., Fang Y., Huang T., Wang S., Zhu Q., Li X., Ge R.S. (2019). Aconitine Inhibits Androgen Synthesis Enzymes by Rat Immature Leydig Cells via Down-Regulating Androgen Synthetic Enzyme Expression in Vitro. Chem.-Biol. Interact..

[99] Wei J., Fan S., Yu H., Shu L., Li Y. (2021). A New Strategy for the Rapid Identification and Validation of the Direct Targets of Aconitine-Induced Cardiotoxicity. DDDT.

[100] Wang X., Yang Z., Zhang Y., Cheng F., Xing X., Wen F., Hu Y., Chen C., Wei B., Bai P. (2022). Tandem Mass Tag Labeled Quantitative Proteomic Analysis of Differential Protein Expression on Total Alkaloid of *Aconitum flavum* Hand-Mazz. against Melophagus Ovinus. Front. Vet. Sci..

[101] Tong H., Chen H., Gong F., Zhong L., Zhu J., Yang S. (2021). Components and Pharmacodynamical Mechanism of Yinfupian Based on Liquid Chromatography-Mass Spectrometry and Proteomics Analyses. Front. Pharmacol..

[102] Liu Y., Lu S., Liu K., Wang S., Huang L., Guo L. (2019). Proteomics: A Powerful Tool to Study Plant Responses to Biotic Stress. Plant Methods.

[103] Robinson J.M., Hodgson R., Krauss S.L., Liddicoat C., Malik A.A., Martin B.C., Mohr J.J., Moreno-Mateos D., Muñoz-Rojas M., Peddle S.D. (2023). Opportunities and Challenges for Microbiomics in Ecosystem Restoration. Trends Ecol. Evol..

[104] Singh M., Kumar A., Singh R., Pandey K.D. (2017). Endophytic Bacteria: A New Source of Bioactive Compounds. 3 Biotech.

[105] Afzal I., Shinwari Z.K., Sikandar S., Shahzad S. (2019). Plant Beneficial Endophytic Bacteria: Mechanisms, Diversity, Host Range and Genetic Determinants. Microbiol. Res..

[106] Tu R., Wang N., Cui L.J. (2022). Composition of Endophytic Bacteria in *Aconitum carmichaelii* Debx. Mod. Chin. Med..

[107] Zou L., Wang Q., Wu R., Zhang Y., Wu Q., Xiong W., Ye K., Dai W., Huang J. (2023). Root Endophytic Bacterial Community Composition of *Aconitum carmichaelii* Debx. from Three Main Producing Areas in China. J. Basic Microbiol..

[108] Hafeez S., Yaqoob S., Magray A.R., Kamili A.N., Ganai B.A. (2023). Molecular Characterization of Fungal Endophyte Diversity Isolated from *Aconitum heterophyllum*: A Critically Endangered Medicinal Plant of Kashmir Himalaya. Int. Microbiol..

[109] Trivedi P., Leach J.E., Tringe S.G., Sa T., Singh B.K. (2020). Plant-Microbiome Interactions: From Community Assembly to Plant Health. Nat. Rev. Microbiol..

[110] Zhao N., Wang G., Norris A., Chen X., Chen F. (2013). Studying Plant Secondary Metabolism in the Age of Genomics. Crit. Rev. Plant Sci..

[111] Singh K.S., Van Der Hooft J.J.J., Van Wees S.C.M., Medema M.H. (2022). Integrative Omics Approaches for Biosynthetic Pathway Discovery in Plants. Nat. Prod. Rep..

[112] Li H., Shi H., Xu P., Yu D. (2022). Metabolomics and Microbiome Reveal Potential Root Microbiota Affecting the Alkaloidal Metabolome in *Aconitum vilmorinianum* Kom. BMC Microbiol..

[113] Owen C., Patron N.J., Huang A., Osbourn A. (2017). Harnessing Plant Metabolic Diversity. Curr. Opin. Chem. Biol..

[114] Wu Y., Zhang C., Huang Z., Lyu L., Li W., Wu W. (2022). Integrative Analysis of the Metabolome and Transcriptome Provides Insights into the Mechanisms of Flavonoid Biosynthesis in Blackberry. Food Res. Int..

[115] Yang H., Wang C., Zhou G., Zhang Y., He T., Yang L., Wu Y., Wang Z., Tang X., Chen G. (2024). A Haplotype-Resolved Gap-Free Genome Assembly Provides Novel Insight into Monoterpenoid Diversification in Mentha Suaveolens “Variegata”. Hortic. Res..

[116] Leng L., Xu Z., Hong B., Zhao B., Tian Y., Wang C., Yang L., Zou Z., Li L., Liu K. (2024). Cepharanthine Analogs Mining and Genomes of *Stephania accelerate* Anti-Coronavirus Drug Discovery. Nat. Commun..

[117] Sun W., Xu Z., Song C., Chen S. (2022). Herbgenomics: Decipher Molecular Genetics of Medicinal Plants. Innovation.

